# Barriers and facilitators to utilisation of public sexual healthcare services for male sex workers who have sex with men (MSW-MSM) in The Netherlands: a qualitative study

**DOI:** 10.1186/s12889-022-13799-1

**Published:** 2022-07-21

**Authors:** Charlotte Merel Marije Peters, Nicole Helena Theodora Maria Dukers-Muijrers, Ymke Joline Evers, Christian Jean Pierre Antoine Hoebe

**Affiliations:** 1 Department of Social Medicine, Care and Public Health Research Institute (CAPHRI), Maastricht University, PO Box 616, 6200 MD Maastricht, the Netherlands; 2 Department of Sexual Health, Infectious Diseases and Environmental Health, South Limburg Public Health Service, PO Box 33, 6400 AA Heerlen, the Netherlands; 3 Department of Health Promotion, Care and Public Health Research Institute (CAPHRI), Maastricht University, PO Box 616, 6200 MD Maastricht, the Netherlands; 4 Department of Medical Microbiology, Care and Public Health Research Institute (CAPHRI), Maastricht University Medical Centre (MUMC+), PO Box 5800, 6202 AZ Maastricht, The Netherlands

**Keywords:** Male sex workers who have sex with men, STI/HIV, Sexual healthcare services, Public health services, Utilisation, STI testing, Sex work stigma, Prevention, Internet fieldwork, Implications for care

## Abstract

**Background:**

Male sex workers who have sex with men (MSW-MSM) are a high-risk group for sexually transmitted infections (STI) including human immunodeficiency virus (HIV). Provision of sexual services by MSW-MSM has shifted to the internet. Consequently, MSW-MSM have become hidden to care for providers of sexual healthcare services (SHS). The aim of this study was to 1) assess characteristics of the MSW-MSM population and 2) assess MSW-MSM’s perceived barriers and facilitators to utilise SHS provided free and anonymously by the public health STI clinic in The Netherlands.

**Methods:**

For this qualitative study, semi-structured individual in-depth interviews were conducted with 20 MSW-MSM who worked home-based in the Dutch province of Limburg. Participants were recruited from November 2018 to June 2019 by purposive sampling until saturation was reached via 1) five websites and smartphone applications commonly used by MSW-MSM, 2) STI clinic, 3) two gay saunas. A theory-informed interview guide was developed including themes such as sexuality, sex work, SHS and barriers and facilitators to SHS utilisation. The interviews’ recordings were transcribed verbatim and thematically analysed by inductive and deductive coding with Atlas.ti 8.

**Results:**

The interviewed MSW-MSM were diverse in age (range: 18 – 66; median: 39.5) and mostly western European (85%). Identified barriers to SHS utilisation were lack of self-identification as homosexual and sex worker, perceived stigma on sex work and MSM, the lack of awareness of SHS and a low STI risk perception. Identified facilitators were trust in and positive attitude towards SHS, awareness of SHS’s anonymous, confidential and free-of-charge nature, high STI risk perception and knowledgeable about STI/HIV. MSW-MSM-identified implications for SHS-providers were promotion of SHS on online MSW-MSM and general platforms (e.g. Facebook), offering one-on-one online and informal communication with an SHS-provider (e.g. STI clinic nurse) and providing STI (testing) information.

**Conclusion:**

The MSW-MSM population’s diversity and identified barriers, facilitators and implications should be taken into account to optimize accessibility and utilisation of SHS for MSW-MSM in Western Europe. SHS-providers could facilitate sex work disclosure by personally asking patients about sex in exchange for money or goods in a non-judgmental manner and explaining the medical relevance of disclosure.

**Supplementary Information:**

The online version contains supplementary material available at 10.1186/s12889-022-13799-1.

## Short summary

Male sex workers who have sex with men (MSW-MSM) have become hidden to care for providers of sexual healthcare services (SHS) due to internet-based work methods. We interviewed twenty MSW-MSM to assess characteristics of the population and MSW-MSM’s perceived barriers and facilitators to utilise SHS. Implications of this study can be used to improve accessibility and utilisation of SHS by MSW-MSM.

## Background

Male sex workers who have sex with men (MSW-MSM) are at high risk for acquiring sexually transmitted infections (STI) including human immunodeficiency virus (HIV). We define MSW-MSM as men who have sex with men in exchange for money or goods. Their risk for acquiring STI is higher (up to 46% positivity) than other known STI risk groups such as female sex workers (FSW) and (non-sex worker) men who have sex with men (MSM) [1–6]. A possible explanation for this is that MSW-MSM involve in high-risk sexual behaviour by frequently engaging in condomless anal sex with clients and using substances, which is reportedly also higher among MSW-MSM compared to MSM and FSW [3, 4, 7, 8]. Multiple studies have shown that a large part (43.4 to 59.6%) of the MSW-MSM population (also) have sex with women, thereby enabling STI/HIV transmission to female sex partners and thus bridging between populations [3, 4, 6, 8]. Therefore, adequate utilisation of sexual healthcare services (SHS) by MSW-MSM is needed from both an individual and a public health perspective.

However, STI/HIV prevention and treatment for MSW-MSM is impeded by reduced access to SHS [9]. In Western countries, the provision of sexual services by MSW-MSM has shifted to the internet in the past years [10, 11]. Soliciting clients independently of a third party from home as an in-call or out-call escort is often referred to as home-based sex work [12]. Internet sites and smartphone applications are widely being used by home-based MSW-MSM to arrange paid sexual encounters [1]. Consequently, MSW-MSM have individualized, become more hidden to providers of SHS and contacting SHS-providers has now become up to MSW-MSM [10]. Globally there is however a lack of knowledge, data and expertise on MSW-MSM who are a neglected STI/HIV high-risk group. Many countries do not routinely collect epidemiological data on MSW-MSM, they have been mostly ignored in the global HIV/AIDS response and limited studies have addressed the needs of MSW-MSM and assessed STI/HIV prevention programs targeting MSW-MSM [1]. The shift towards an internet-based work method has also affected the scientific rigour of research by complicating the identification, sampling and assessment of MSW-MSM [1, 13]. Furthermore, studies focused on MSW-MSM mainly include those who professionally sell sex and are thus often not representative of the MSW-MSM population who mostly sell sex with a dating and hustle motivation [14, 15]. For MSW-MSM to adequality utilise SHS and to increase accessibility of SHS there is a need to identify facilitators and barriers to SHS utilisation by MSW-MSM in Western Europe. However, limited research has been conducted among MSW-MSM in a similar research context to identify these facilitators and barriers. A few barriers to SHS utilisation by MSW-MSM in the Netherlands have been identified, namely a perceived double stigma on sex work and homosexuality, a lack of trust in healthcare providers and a need to stay hidden from institutions like the police , tax authorities and the municipality [10, 16, 17]. In the Netherlands sex work is legal, however, municipalities often do not allow sex work from home or will not issue a permit to home-based sex workers. Home-based sex workers are consequently often illegally working as a sex worker, risk losing their income and residence and are not eager to contact the police and other governmental institutions [12]. Also, this could possibly negatively influence the utilisation of SHS and disclosure of sex work at SHS. The hidden character of the MSW-MSM population together with perceived barriers emphasize the complexity of improving SHS utilisation for this population and the need for a tailored approach for MSW-MSM [1]. However, such an approach has yet to be developed by SHS. Thus, the main objectives of this study were to 1) assess characteristics of the MSW-MSM population and 2) assess MSW-MSM’s perceived barriers and facilitators to utilise the STI clinic’s SHS in the Netherlands.

## Methods

### Study design

To assess the perspectives of MSW-MSM concerning sex work and SHS, a qualitative descriptive study was conducted for which semi-structured in-depth interviews were held. The Health Belief Model (HBM) [18] describes that health behaviour is determined by perceived severity and perceived susceptibility of the health issue and benefits and barriers of the health behaviour and a cue to action could provide an extra stimulant. Perceived (social) norm, attitude and knowledge might also play a role as described in the Reasoned Action Approach Model (RAAM) [19]. We assessed the health behaviour of SHS utilisation by MSW-MSM partly by assessing determinants of health described in HBM and RAAM [18, 19]. The study context is described in Additional file 1.

### Participant selection

Participants were eligible for inclusion in this study if 1) biologically of the male sex, 2) aged 18 years and above, 3) had at least once sex with a man in exchange for money or goods in the past 6 months, 4) the sex work has taken place in the Dutch province of Limburg. The participants were recruited through purposive sampling via three routes: the STI clinic of South Limburg, internet fieldwork and two male saunas in the region of South Limburg. We have put more emphasis on recruitment through IFW in an effort to recruit MSW-MSM who had not been tested and reached before by the STI clinic. The recruitment posters and the information letters were developed in Dutch, English and German to also attempt recruitment of non-Dutch sex workers. Recruitment posters were hung up at the STI clinic’s waiting rooms and in two gay saunas. Additionally, during STI consultations nurses recruited MSW-MSM and their peers. Four online platforms commonly used by MSW-MSM (Grindr, Bullchat, Boys4U and Kinky) were used for participant recruitment through internet fieldwork. Both an active and passive approach were used for recruitment. With the active approach, a recruitment message was sent to profiles likely of MSW-MSM. With the passive approach, an STI clinic nurse was present on the online platforms and the recruitment poster was used as the profile picture. Recruitment continued until data saturation was reached i.e. until information redundancy regarding the topic of SHS utilisation occurred. The recruitment process took place from November 2018 to June 2019. In total 29 participants were recruited, of which we interviewed 20 participants, 7 were no-shows and 2 were excluded due to not wanting to comply with the interview criteria such as interview location and due not being a sex worker but a sex work client. Of the 20 interviewed participants, 14 were recruited through internet fieldwork, 3 through the STI clinic and 2 through the male saunas.

### Data collection

A theory-informed interview guide was developed based on relevant theoretical constructs from the Health Belief Model (HBM) [18] and the Reasoned Action Approach Model (RAAM) [19], concepts retrieved from literature and themes of interest. Themes included were STI and STI testing behaviour, sexuality and sex work, experienced and perceived stigma, attitude, perceived benefits and perceived barriers towards SHS utilisation and STI clinic and needs for SHS.

The interviews were approximately 1.5 hours and were conducted face-to-face at a location and time of the participant’s preference. Of the 20 interviews (17 in Dutch and 3 in English), 15 were held at the STI clinic and 5 at the participant’s home. No non-participants were present at the interviews. Both oral and written informed consent were obtained. Participants received 50 euros cash as compensation for their time and a goodie bag. The goodie bag included condoms, lubricant, information leaflets on, amongst others, STI, chemsex, sex work and support organisations as well as sweets and personal care products. As part of our regular outreach care, participants were offered the possibility to do an STI test, receive a hepatitis B vaccination and information at the interview location or to make an appointment at the STI clinic.

The interviews were recorded by a voice recorder, transcribed verbatim and anonymized. Field notes were made after the interview regarding the general impression of the interviews. Transcripts were not returned to the participants for comments, however, one participant was sent his interview transcript on request.

### Data analysis

For this study we performed a thematic analysis, allowing us to identify, analyse, organize and report themes in the data in detail [20]. To ensure the quality of our thematic analysis, we used the 15-point checklist of criteria for good thematic analysis developed by Braun and Clarke [20].

All transcripts were imported into the software Atlas.ti 8. We used a hybrid process of inductive and deductive coding. An initial coding structure was developed based on concepts and themes from the interview guide. Emergent codes were added based on the inductive coding strategy of active reading. Coding was done by the researcher who had also conducted the interviews and who is fluent in Dutch and English. The first two coded transcripts were reviewed by a senior researcher and discussed until a coding consensus was reached. Due to previous coding experience in a similar research topic, discussing two coded transcripts proved sufficient to establish a coding consensus. The coding of the remaining transcripts was continued by the researcher who had also conducted the interviews. Finally, the code descriptions were discussed with the research team for interpretation purposes.

This study is reported in accordance with the consolidated criteria for reporting qualitative studies (COREQ) checklist [21].

### Ethical considerations

Ethical approval was provided by the Ethical Review Committee Psychology and Neuroscience of Maastricht University (reference number OZL 188_10_02_2018_S13).

## Results

A diverse group of twenty male sex workers who have sex with men participated in this study (Table 1).

**Table 1:** Socio-demographic characteristics of the study population

Characteristic	N (%)
Biological gender	
Male	20 (100)
Gender identity	
Male	18 (90)
Female	2 (10)
Average age (in years)	39.9 (Range: 18 – 66; Median: 39.5 )
Country of birth^a^	
The Netherlands	14 (70)
Western Europe other	3 (15)
Eastern Europe	2 (10)
South America	1 (5)
Level of education^b^	
Low	2 (10)
Medium	10 (50)
High	8 (40)
Employment besides sex work	
Employed	11 (55)
Unemployed	9 (45)
Duration of sex work (range)	3 months – 32 years
Relationship status	
In a relationship	7 (35)
Not in a relationship	13 (65)
Sexual preference	
Homosexual	11 (55)
Bisexual	7 (35)
Heterosexual	2 (10)

^a^According to United Nations Statistics Division geographic regions [22]

^b^Level of education was categorized into low: elementary, pre-vocational secondary; medium: senior general secondary, pre-university, secondary vocational; high: higher professional, university

All participants were biologically of the male gender. Some of those unemployed besides their sex work were still in education, unable to work due to medical reasons or retired. The duration of sex work ranged from 3 months to 32 years, however sex work was often done with intermissions. At the time of the interview, 7 participants were in a relationship, of which 2 had a relationship with a man. Nearly half (45%) of the participants did not identify as homosexual. Those who identified as heterosexual had a male gender identity.

Identified barriers and facilitators to SHS utilisation by MSW-MSM and implications to increase the accessibility and utilisation of SHS by MSW-MSM are presented in Table 2 and discussed below per the following themes: sex work and sexuality, shame and stigma, sexual healthcare services, STI and STI testing and increasing SHS accessibility and utilisation by MSW-MSM. Illustrative quotations of the themes identified in this study are provided in Table 3. Themes related to the context of the study population, i.e. start and reason of sex work and social support, are presented in Additional file 1.

**Table 2:** Identified barriers and facilitators to SHS utilization by MSW-MSM and implications for SHS

Barriers	Facilitators	Implications SHS
**Sex work and sexuality**		
Lack of identification as sex worker	- Identifying as sex worker - Willingness to disclose sex work	- Promotion of MSW-MSM SHS and IFW^1^ on online platforms commonly used by MSW-MSM - Using more neutral, non-stigmatizing terms of sex work in communication messages - Informing on the relevance of disclosure for the provision of tailored SHS
Lack of social support regarding sex work		- Providing social support in consultations - Permanent contact person - Linking MSW-MSM, organizing peer group meetings
Lack of identification as homosexual		- Promotion of MSW-MSM SHS on more general platforms, e.g. social media, TV - Communication not specifically focused on LGBTQIA+ community, both in communication message and visualization
**Shame and stigma**		
Experiencing shame and (self-)stigma MSM	Not experiencing shame and (self-)stigma MSM	- Communication message: stressing anonymous and confidential nature of SHS for MSW-MSM and using non-stigmatizing language - Providing home self-sampling STI kits
Experiencing shame and (self-)stigma sex work	Not experiencing shame and stigma sex work	- Communication message: stressing anonymous and confidential nature of SHS for MSW-MSM and using non-stigmatizing language - Providing home self-sampling STI kits
Perceived negative social norm towards MSM and sex work		- Personal contact with a nurse - Informal and positive communication style - Communication message: stressing anonymous and confidential nature of SHS for MSW-MSM, non-judgmental environment of STI clinic and professional experience in MSM and sex work field - Providing home self-sampling STI kits
Fear of stigmatization towards MSM and sex work		- Personal contact with a nurse - Informal and positive communication style - Communication message: stressing anonymous and confidential nature of SHS for MSW-MSM, non-judgmental environment of STI clinic and professional experience in MSM and sex work field - Providing home self-sampling STI kits
**Sexual healthcare services (SHS)**		
Negative attitude STI clinic	Positive attitude STI clinic	- IFW to promote MSW-MSM SHS and counsel MSW-MSM on online platforms commonly used by MSW-MSM - Personal contact with a nurse - Informal and positive communication style - Communication message: stressing anonymous, free and confidential nature of SHS for MSW-MSM
Low awareness of SHS STI clinic	High awareness of SHS STI clinic	- Promotion of MSW-MSM SHS through active and passive IFW on online platforms commonly used by MSW-MSM - Promotion of MSW-MSM SHS on more general platforms, e.g. social media, TV - Promotion of MSW-MSM SHS in gay saunas and other popular gay meeting places - Communication message: stressing anonymous, free and confidential nature of SHS for MSW-MSM
Lack of trust in STI clinic	Trust in STI clinic	- Personal contact with a nurse - Informal and positive communication style - Communication message: stressing anonymous, free and confidential nature of SHS for MSW-MSM and explaining term STI clinic
Lack of awareness of anonymity and confidentiality of the STI test and -clinic	Awareness of anonymity and confidentiality of the STI test and -clinic	- Promotion of MSW-MSM SHS through active and passive IFW on online platforms commonly used by MSW-MSM - Promotion of MSW-MSM SHS on more general platforms, e.g. social media, TV - Promotion of MSW-MSM SHS in gay saunas and other popular gay meeting places - Communication message: stressing anonymous and confidential nature of SHS for MSW-MSM
Disclosure concerns: - having to disclose sex work to an unknown healthcare professional - fear of judgement - lack of understanding of (medical) relevance of disclosure	- Willingness to disclose sex work - Explicitly and personally being asked for sex work status in a non-judgmental manner - Aware of the medical relevance of disclosing sex work	- Personal contact with a nurse - Informal and positive communication style - Communication message: stressing anonymous and confidential nature of SHS for MSW-MSM, non-judgmental environment of STI clinic and professional experience in sex work field - Informing on the relevance of disclosure for the provision of tailored SHS - Providing home self-sampling STI kits
Practical aspects of getting tested: - calling to make an appointment - freeing up time to get tested - distance and transportation to the STI-clinic		- Providing home self-sampling STI kits - Providing user-friendly appointment system including possibility to make appointments online - Personal reminders
**STI and STI testing**		
	Positive attitude STI test	- Providing general STI test procedure information through online platforms commonly used by MSW-MSM
Positive perceived norm STI test - Social support STI test - Modelling STI test	Negative perceived norm STI test	Normalizing STI testing by promotion of STI testing on both general platforms and online platforms commonly used by MSW-MSM
Low STI risk perception: - Low perceived susceptibility STI - Low perceived severity STI	High STI risk perception - High perceived susceptibility STI - High perceived severity STI	- Providing STI risk information through online platforms commonly used by MSW-MSM - IFW to promote MSW-MSM SHS and counsel MSW-MSM to provide personal risk information on online platforms commonly used by MSW-MSM - Communication message: Stressing own health benefits, responsibility towards sexual partners and preventing infecting them with STI (gain frame)
Fear of results STI test	- Understanding of health benefits STI test - Feeling sense of social responsibility to not transmit STI to sex partners	- Providing general STI information through online platforms commonly used by MSW-MSM - IFW to promote MSW-MSM SHS and counsel MSW-MSM to provide tailored information on online platforms commonly used by MSW-MSM
Fear of possible costs associated with the STI test	Awareness of available free SHS at STI clinic	- Promotion of MSW-MSM SHS and active and passive IFW on online platforms commonly used by MSW-MSM - Promotion of MSW-MSM SHS on more general platforms, e.g. social media, TV - Promotion of MSW-MSM SHS in gay saunas and other popular gay meeting places - Communication message: stressing free nature of SHS for MSW-MSM
Fear of needles		- Providing general STI test procedure information through online platforms commonly used by MSW-MSM - IFW to promote MSW-MSM SHS and counsel MSW-MSM to provide tailored information regarding the STI test procedure on platforms commonly used by MSW-MSM
Perceived stigma on STI test		- Normalizing STI testing by promotion of STI testing on both general platforms and online platforms commonly used by MSW-MSM - Providing home self-sampling STI kits
Lack of STI knowledge - Lack of understanding of STI and STI test - Not feeling a sense urgency to get tested for STI due absence of symptoms	Knowledgeable about STI - Understanding health benefits of getting tested for STI - Understanding of STI and their (absence of) symptoms	- Providing general STI and STI test information through online platforms commonly used by MSW-MSM - IFW to promote MSW-MSM SHS and counsel MSW-MSM to provide tailored information regarding the STI and STI test on platforms commonly used by MSW-MSM

^1^IFW: internet fieldwork

**Table 3:** Illustrative quotations per theme

Theme		Participant characteristics	Quotation
**Shame and stigma**			
Identification sex worker		Participant 16 49 years old	“At least I wasn't on it all day, not at all. Far, far from even. So it's not something you need to pay your bills. And with prostitution you have a completely different view of- Prostitution is just something completely different. Are you, there you are just looking for customers who pay continuously. So and that's not how I saw it. It was actually more my pleasure - And that's why I don't see it as sex work."
Identification homosexual		Participant 18 48 years old	“I am bisexual, but uh, I could never live with a man. Is purely sexual, yes.”
**Shame and stigma**			
Shame and (self-)stigma sex work	Not experiencing shame and (self-)stigma	Participant 15 40 years old	“Never, no. I am not ashamed for things I do. And I- Not ashamed, not even what you or other thinks about me because I am a prostitute. I am because I have to be. That's my story, happen like this, was to be like this. I am like this. Shoot me if you don't like it.”
	Experiencing shame and (self-)stigma	Participant 12 37 years old	“Normally it's a bit embarrassing, in that sense. A little though. That's um, still learning to cope better. 'Cause, yes it's me. So you have to yes, otherwise you will get depression.”
Shame and (self-)stigma MSM		Participant 18 48 years old	“That’s what I don’t know. Is this an addiction? Is this an illness? Is this a- are these the genes of my parents?”
Experienced stigmatization	MSM	Participant 8 35 years old	“Here’s the situation. I’m gay. And I remember, my mother don’t talk to me for three months. Three months. She don’t cook for me. She don’t clean eh, eh, clean my clothes. Anything.”
	Sex work	Participant 19 35 years old	“Because as sex worker you are like nothing in the face of other people.”
**Sexual healthcare services (SHS)**			
Attitude SHS		Participant 7 39 years old	“Always very relaxed and very helpful and when I have questions, it goes really smoothly. I do have a familiar feeling, though. I think if there had to be something, I could ask or say anything or something. Absolutely, I don't have to hide anything."
Awareness available SHS		Participant 16 49 years old	“I don't know if there are any costs involved. Now you're saying that it's free ehm, also that eh, that's very ehm-… I think it, I don't know exactly what that test entails. Do you need to draw blood or uh, me, what does that mean?”
Need for sexual health information		Participant 6 55 years old	“You see, you don't like to talk about, what is gonorrhoea? What is syphilis? Yes, maybe you could have a little more info on that. Maybe it's on the website? I'm not sure.”
Disclosure sex work		Participant 7 39 years old	“You have to tick that box on the list but I have never done that. No I have never. Because I thought, otherwise they might ask questions or this or that. But I would do that now, after this conversation I think. I think so. Actually yes.”
**STI and STI testing**			
Attitude STI test		Participant 3 44 years old	“It’s normal, so not a problem. It's for your own health, right?”
Perceived (social) norm STI test		Participant 5 46 years old	“I think they're thinking too lightly about it, about the necessity of it. Well, I hardly discuss it, this is just the impression I have.”
Perceived susceptibility STI		Participant 7 39 years old	“There is always a risk of course, but I don't think it's that big because I'm always very careful. Okay, sucking may be done without a condom, but the fucking is always with. Well, with one exception.”
Perceived severity STI		Participant 20 24 years old	“Well, this might sound stupid, but so to say, most of them can be treated with a shot or with antibiotics, so I don't lose any sleep over that. But if it really were HIV…that’s my biggest fear I guess”
**Increasing SHS accessibility and utilisation by MSW-MSM**			
Suggestions to increase accessibility for MSW-MSM	Promotion of SHS and internet fieldwork on online platforms commonly used by MSW-MSM	Participant 14 18 years old	“I'm actually just thinking through Grindr or through Bullchat or something. Just through the apps, because otherwise you don't really come into contact with them. … I do think that you should simply address via an account, guys, eh, these are the options, we can offer you this, eh, think about it.”
	Provision of STI home-sampling kits	Participant 16 49 years old	“I think for a lot of people it would be easier if they could do some kind of home test. I think that will convince a lot of people… I think if someone is in a familiar environment, it's easier.”
Communication towards MSW-MSM		Participant 18 48 years old	"No, I just think that the main point should be that it’s anonymous, that a lot of men then will give in."

### Sex work and sexuality

#### Identification sex worker

More than half of the participants did not self-identify as a sex worker, mainly due to it not being their main income and enjoying the sex which makes it a win-win situation. A few participants never thought of it as sex work or also didn't want to give it a label.

A number of participants did self-identify as sex workers, mainly due to being paid to perform sexual acts and due to advertising on websites.

#### Identification homosexual

More than half of the participants self-identified as homosexual, followed by a largely bisexually identifying group. Some of these bisexual participants noted that they did not have romantic, but purely sexual feelings towards men.

Few other participants with the male gender identity identified as heterosexual and were attracted to the female gender.

### Shame and stigma

#### Shame and (self-)stigma sex work

Many indicated not to experience shame for their sex work. According to these participants sex work happens anyways, one has to accept who they are and home-based instead of street-based sex work results in not experiencing shame.

Some were ashamed of their sex work, because they did not perceive sex work as a normal and real job and they were still working on self-acceptance. Sometimes they mostly felt shame for and towards their family and partner. They contemplated why they “lower” themselves to the level of sex work and if sex work is morally reprehensible. Sex work sometimes lowered self-image and self-esteem and would cause participants to feel objectified. Due to the sex work, one participant also felt useless to society and depressed.

#### Shame and (self-)stigma MSM

Part of the participants experienced feelings of shame and self-stigma for their sexuality and/or having sex with men. They mostly struggled with self-acceptance of their bisexuality, especially their sexual feelings towards men. They perceived their sexual attraction towards men as an illness, deviant and as not matching the requirements of a ‘standard man’. Due to having sex with men, some participants had an anticipated fear of their social environment’s reaction to and consequences of coming out, an anticipated fear of stigmatization, a feeling of not being socially accepted and a lowered self-image.

Some participants indicated to not be ashamed anymore, but to have felt shame in the past for being an MSM. Half of the interviewed MSW-MSM however indicated that they don’t experience shame for their homosexuality and/or having sex with men.

#### Experienced stigmatization

Many had experienced stigma, negative and stigmatizing responses, towards their homosexuality from their social environment, family members, friends, acquaintances and colleagues.

Some also experienced stigma towards their sex work and experienced a noticeable taboo on sex work in society. Often they were treated in a diminishing way and called diminishing names such as “whore” and “brown worker”. These stigmatizing experiences gave them a feeling of inferiority.

### Sexual healthcare services

#### Attitude SHS

The vast majority of the participants who had experience with the STI clinic’s SHS, approximately three quarters, had a positive attitude towards it. They experienced the STI clinic as an accessible, non-judgmental, and trustworthy environment. The easiness of doing an STI test was frequently mentioned as well as the pleasantness of the anonymous and free-of-charge services. Several participants who had online communication with an STI clinic nurse on an online MSW-MSM platform also had a positive attitude regarding online counselling.

One participant had a positive attitude towards the STI clinic’s SHS in the past, until misinformation was spread, after which he never went to the STI clinic again. A positive attitude was regained due to the positive interview experience. Those participants who had no experience with the STI clinic’s SHS either said not to have any perceptions due to the lack of experience with the STI clinic’s SHS or had a positive attitude due to the positive interview experience despite having a negative attitude before.

#### Awareness available SHS

More than half of the participants had moderate to poor awareness of the SHS available for sex workers at the STI clinic. They were mostly aware of the possibility to get tested for STI, but many were not aware of the available SHS specifically for sex workers and services being free of charge, confidential and/or anonymous. Only a few were aware of PrEP care, which was newly available at the time of the interview.

Those aware of the STI clinic's SHS were made aware through various channels, most frequently via the internet, as well as via medical professionals, the STI clinic’s (online) outreach and gay meeting locations.

#### Need for sexual health information

Some of the participants indicated to still need information about sexuality and STI, the confidentiality of the STI clinic and hepatitis B. Providing general STI information via online channels, such as Grindr, Instagram and the STI clinic website, also appeared to be an important need.

A majority of participants missed information as novice sex worker. The information needs were mainly related to aspects of sex work, such as risk avoidance and safety during sex work e.g. tips for recognizing risky customers, tips for avoiding STI risks and tips to promote general safety during sex work as well as consequences of sex work, mental health and general STI, SHS and hepatitis B information.

#### Disclosure sex work

Part of MSW-MSW purposefully did not disclose their sex work to the STI clinic. Mostly they did not view disclosing their sex work as relevant for the STI test. Additionally, having to explain the sex work, being scared of questions, shame and fear of judgement were factors that contributed to not disclosing their sex work. A few of them indicated that they would disclose their sex work if they were asked in person.

Some participants indicating that they would disclose the next/first time visiting the STI clinic seemed mostly convinced to disclose after having the interview and knowing the STI clinic is non-judgmental and knowledgeable on the topic of sex work.

### STI and STI testing

#### Attitude STI test

The majority had a positive attitude towards STI tests. Many felt that testing for STI is normal and benefits their health. Even though a couple of MSW-MSM were scared of needles, they still had a positive overall attitude towards STI tests. Despite a positive attitude towards STI tests, a participant did experience small practical barriers to get tested. A few participants had a negative attitude towards STI tests. Given reasons were negative prior experiences, having to expose their sex life and experiencing stress awaiting the test results. However, despite their negative attitude towards STI tests they felt a need to get tested for STI and were aware of its importance. One of the participants said not to have an attitude towards STI testing because he was unaware of what an STI test entails.

#### Perceived (social) norm STI test

The perceived social norm of STI tests is positive according to half of the participants. Their environment was supportive of STI testing and some had friends and (sex) partners who would get tested regularly. The social norm of STI tests is negative, especially for sex workers, according to part of the study population. Sex workers allegedly experience barriers to get tested and view testing as an invasion of privacy and not important. Additionally, heterosexual friends did not perceive STI testing as important and were too coy to get tested. However, many were not aware of the social norm for STI tests. They said the topic of STI tests would not be discussed in their social environment and seemed to be a taboo topic.

#### Perceived susceptibility STI

Many had a low perceived susceptibility of STI. They estimated their risk of contracting an STI from 0 – 25%. Reasons being having sex with condoms, being careful and aware of risks and taking precautions, some not having receptive anal sex and having condomless sex with a regular group of people. However, many of these participants also had oral sex without a condom and sometimes had condomless anal sex with men they found attractive. They could not exclude the possibility of contracting STI and felt that the high number of sex partners increased their STI risk. One participant did not feel at risk due to not having contracted an STI for years and not perceiving the anus as a sexual organ.

Part had a high perceived susceptibility of STI and estimated their risk of contracting an STI from 25 – 100%. Reasons being their high number of sexual partners, having condomless oral and anal sex, the possibility of condom failure and having been warned for an STI multiple times. Among these participants were those who did and those who did not portray sexual risk behaviour. They felt that as a sex worker and having sex in their private life as well, they were at high risk for an STI and contracting an STI wouldn’t always be preventable.

#### Perceived severity STI

The majority perceived contracting an STI as mildly severe to severe. Having an STI would bother them and they would feel responsible for not being careful enough. Some would worry about how they contracted the STI and how to prevent it in the future. Getting treatment as soon as possible was considered important. Often MSW-MSM were mostly worried about having to warn their sex partners and/or partner they are in a relationship with, concerned about how this will change their relationship, as well as practical implications for their sex work. Those who did not perceive contracting an STI as severe, about one-fourth, had the same concern. They however perceived the health impact of an STI as minor, since simply taking medicine would cure their STI. The MSW-MSM sometimes seemed to not see HIV as part of STI. HIV was perceived as more severe than STI in general. Some dreaded possibly contracting HIV and feared the stigma that comes along with it. HIV was perceived so severely because of its deadly consequences, even though one is aware that HIV is well treatable in the Netherlands nowadays.

#### Perceived barriers STI test

Many experienced barriers to getting tested for STI, as presented in Table 2. The barriers often did not cause them not to get an STI test at all, but did make getting tested for STI more challenging.

### Increasing SHS accessibility and utilisation by MSW-MSM

#### Suggestions to increase accessibility for MSW-MSM

The following suggestions were made by the participants to increase SHS accessibility and consequently MSW-MSM SHS utilisation:

- Promotion of the STI clinic’s SHS on all online platforms (websites and apps) commonly used by MSW-MSM for contacting customers. Promotion can be done in the form of a banner or advertisement, an information link about the STI clinic or by doing active and passive internet fieldwork. It was also regularly suggested to use both passive and active internet fieldwork approaches, i.e. promotion in the form of a banner or advertisement, being available for sexual health related questions in chatrooms as well as actively sending messages to possible sex workers. Sex workers can be recognized on online platforms by a money sign, money bag, diamond or “pay” in their name.
- Promotion of SHS on general (social) media platforms e.g. on Instagram, Facebook, TV, radio and door-to-door promotion. This approach would reach more hetero and bisexually identifying MSW-MSM.
- Promotion of SHS at gay saunas and other gay meeting locations (i.e. parties, gay cinemas, parking lots). This would mainly be in the form promotional posters or visiting the location for STI testing and counselling.
- Building trust between the STI clinic and MSW-MSM. Trust could be increased through informal, friendly and clear communication at the MSW-MSM language level. Professional language allegedly increases the distance between the STI clinic and MSW-MSM. Providing an explanation about the STI clinic for non-Dutch MSW-MSM was also mentioned as important to avoid mistrust.
- Building a personal relationship with an SHS-provider e.g. STI clinic nurse. This could make MSW-MSM feel more at ease and facilitate trust-building.
- The provision of STI home-sampling kits. Some MSW-MSM would find it convenient, easy and pleasant to obtain the materials for the STI test in their own safe environment. Participants suggested either sending the kit to their home address or being able to pick the kit up at a pick-up point.
- Providing an accessible appointment system. This includes the option to make an appointment online and to be able to complete the medical questionnaire online.
- Personal WhatsApp contact with a nurse. Participants mentioned this would remove experienced barriers of having to call for an STI test appointment, a lack of trust and familiarity and disclosure concerns.
- Receiving a personal reminder to make an appointment for an STI test. This suggestion was said to increase STI testing behaviour, since this strategy worked well for a participant in the past.

#### Communication towards MSW-MSM

Many participants highlighted the importance of mentioning the anonymity of STI tests and the free-of-charge SHS in communication towards MSW-MSM.

With regard to language-use, it was considered important that it is not long-winded and patronizing but clear, informative, informal, positive and non-stigmatizing. Several times it was suggested to emphasize the importance of testing for your own health, but also out of responsibility for your sex partners. The communication and visuals should not seem specifically focused on sex workers and the LGBTIQ+ community. One participant however noted that due to the diversity of the MSW-MSM population there will not be one suitable message for the entire MSW-MSM population.

### Determinants of SHS utilisation

**Fig. 1:**
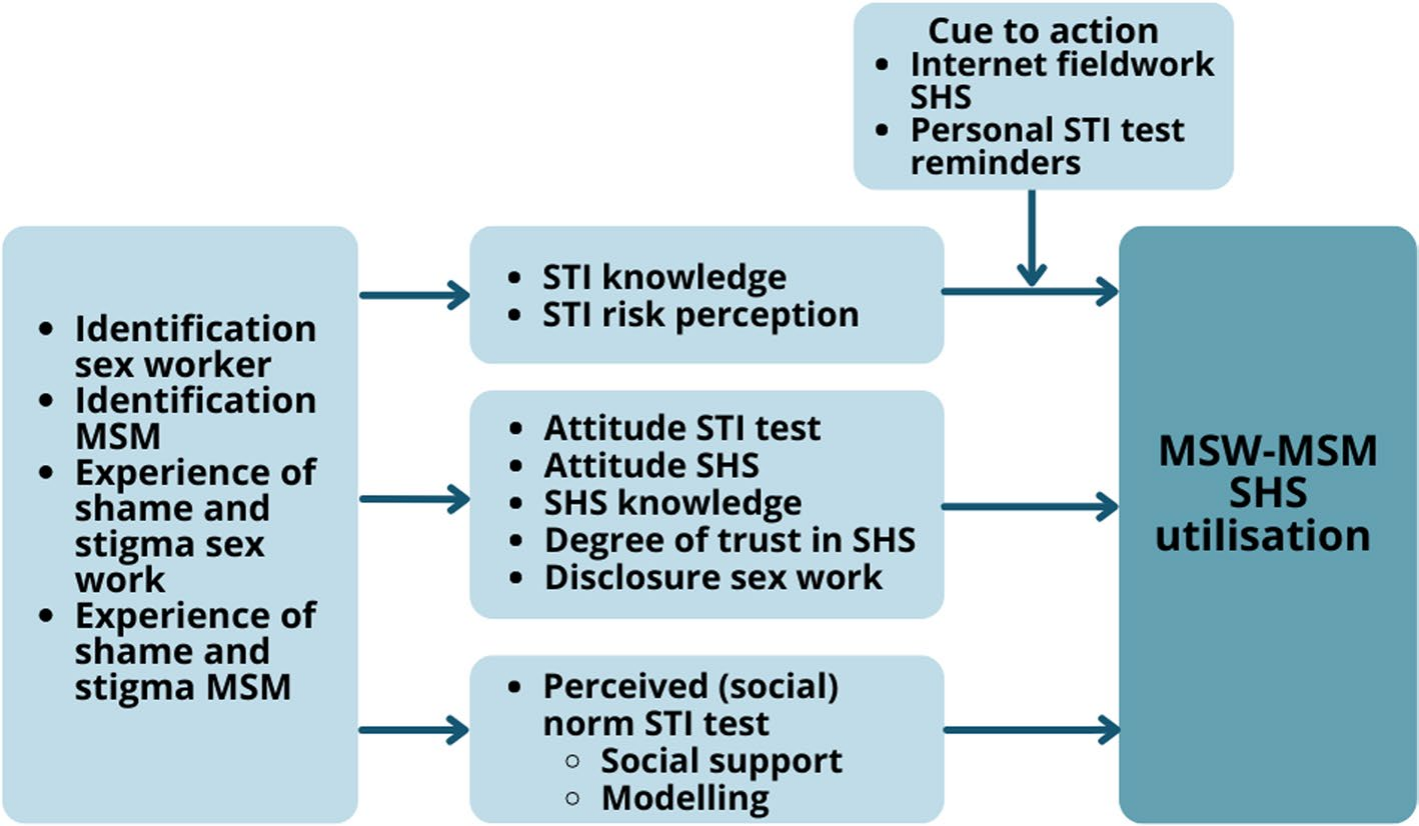
Identified key factors influencing MSW-MSM SHS utilisation based on HBM and RAAM. ^i^HBM: Health Belief Model; RAAM: Reasoned Action Approach Model. ^ii^Constructs and pathways are derived from the Health Belief Model (HBM) and Reasoned Action Approach Model (RAAM). Identification sex worker, identification MSM, experience of shame and stigma sex work, experience shame and stigma MSM are (social)demographic and psychological characteristics derived from both HBM and RAAM; STI risk perception, cue to action, perceived barriers and advantages health behaviour: attitude STI test, attitude SHS, degree of trust in SHS, disclosure sex work are derived from HBM; SHS knowledge, perceived (social) norm STI test, social support, modelling (of health behaviour) are derived from RAAM

This study confirmed that several determinants of health behaviour from HBM [18] and RAAM [19] influence the utilisation of SHS, more specifically STI/HIV testing (see Fig. 1).

## Discussion

This study has been able to assess characteristics of the MSW-MSM population, identify perceived barriers and facilitators of MSW-MSM to SHS utilisation and formulate implications to improve accessibility and utilisation of SHS for MSW-MSM. To our knowledge, this is the first qualitative study in Western Europe that has addressed barriers, facilitators and implications of SHS utilisation for MSW-MSM. We established several barriers to SHS utilisation, namely the lack of (self-)identification as sex worker and as homosexual, which consequently hampers disclosure to SHS-providers and addressing their SHS needs, lowers STI risk perception and causes SHS communication to not appeal to MSW-MSM. By not identifying as sex worker the participants in this study also seemed to feel less at risk and susceptible of STI, which negatively influenced their STI risk perception and SHS utilisation. Further identified barriers were perceived stigma on both sex work and MSM and a low STI risk perception. A lack of STI information acted as a barrier as well e.g. not feeling a sense of urgency to get tested due to not experiencing symptoms. Similar results were found in a study conducted among street-based MSW-MSM in the United States and Germany, identifying misperception of all STI being symptomatic, a lack of concern about STI (besides HIV), a perceived low risk of HIV as barriers to STI/HIV testing [23, 24]. There was also a lack of awareness of SHS available especially for sex workers, as well as the anonymous, confidential and free-of-charge nature of the SHS. A study in the United States confirms that concerns of confidentiality and privacy were a central barrier to SHS utilisation by MSW-MSM [13].

Factors facilitating SHS utilisation were trust in and positive attitude towards the STI clinic, awareness of SHS anonymity, confidentially and free-of-charge nature, a high STI risk perception and being knowledgeable about STI. Implications for SHS to improve accessibility and utilisation by MSW-MSM were the promotion of SHS both on online MSW-MSM platforms through internet fieldwork and on general platforms. One-on-one online and informal communication with SHS-provider, e.g. STI clinic nurse, and providing STI information are implications for communication towards MSW-MSM. The results of this study provide important knowledge on the understudied STI/HIV high-risk group of MSW-MSM. The results can be used to improve accessibility and utilisation of SHS by MSW-MSM and to develop tailored healthcare strategies and STI/HIV prevention programs. Consequently, this could decrease the existing health inequity for MSW-MSM, lower the burden of STI among this population and improve individual and public sexual health status.

### Implications for SHS-provider

We found that there was a lack of self-identification as sex worker, which was previously in a Dutch study argued to be partly caused by different sex work motivations [10]. Aside from the lack of identification as a sex worker, we found that almost half of our MSW-MSM study population did not identify as homosexual which is in line with the findings in multiple studies [3, 4, 6, 8, 15, 24]. Furthermore, this study identified that some MSW-MSM had disclosure concerns and willingly did not disclose their sex work to the healthcare professional and SHS-providers. Medical mistrust and perceived stigmatisation by healthcare providers were previously found to create barriers for sex work disclosure, with disclosure concerns and non-disclosure of sex work consequently impeding SHS access for MSW-MSM in the United States and Canada [25, 26]. Additionally, fewer MSW-MSM reported disclosure and more reported mistrust of healthcare providers compared to non-sex worker MSM, indicating that MSW-MSM are a different population from non-sex worker MSM who need a tailored healthcare approach [25]. Perceiving stigma on sex work and MSM was also identified as a barrier to SHS utilisation. Studies confirm that stigma on both homosexuality/bisexuality and sex work would cause the MSW-MSM to not want to disclose and be known as a sex worker and MSM by institutions and that these are important barriers to healthcare access [10, 24, 26, 27]. Several barriers identified in this study, such as disclosure concerns and perceived stigma, were also identified for FSW [28]. Previous research in the Netherlands has described that MSW-MSM also want to stay hidden from government institutions due to the illegal nature of their sex work [16, 17], this however has not specifically come forward in our study.

An important implication of this study is for SHS to conduct internet fieldwork to promote MSW-MSM SHS and counsel MSW-MSM to provide personal risk information on online platforms commonly used by MSW-MSM. The potential of this implication is highlighted in a previous study that suggests that online MSW-MSM platforms and communities could provide a new manner of sexual health promotion and have a great potential to reach MSW-MSM [1, 29].

We have previously addressed that MSW-MSM are considered hidden to care. Due to the lack of identification as sex worker and due to disclosure concerns, part of the MSW-MSM population however is likely to visit the SHS as a ‘regular’ MSM and thus is underregistered as sex worker. We established that SHS-providers could facilitate disclosure by explicitly and personally asking patients about sex in exchange for money or goods in a non-judgmental manner and explaining the medical relevance of disclosing sex work. The reported perceived stigma and disclosure concerns and its previously found consequences highlight the importance of the identified facilitator of trust in the SHS-provider. A study in the United States confirms the importance of a non-judgmental attitude and trustworthiness of SHS [30]. It seems essential for healthcare providers to adopt a professional norm when providing care to sex worker patients, in order to avoid stigmatising experiences and create a non-judgmental environment and trust. While PrEP was newly available at the time of the interviews and thus not thoroughly discussed, SHS-providers should consider MSW-MSM as a highly important target population for PrEP. Furthermore, in previous research it was argued that while STI information may be widely available on the internet, MSW-MSM need help navigating which sites offer correct and trustworthy information [29]. Providing direct contact information of the SHS-provider, such as a mobile telephone number, could help to facilitate informal one-on-one contact, provision of tailored STI information and trust-building.

### Strengths and limitations

This study has several strengths. This study responds to the global call to action for research on MSW-MSM and their sexual healthcare needs in an attempt to reduce the knowledge gap of this population [9]. Furthermore, our recruitment method managed to successfully recruit participants from the MSW-MSM population to provide an in-depth view of SHS utilisation barriers and facilitators. This shows that with extensive and tailored efforts it is possible to reach the hidden to care MSW-MSM population. We also managed to create a non-judgmental and open interview setting, with many participants being grateful for the conversation and willing to discuss other sexual issues or traumas after the interview. This study also has limitations. Despite efforts to recruit a diverse and representative study population through the purposive sampling method, several socio-demographic characteristics such as low education level and non-Dutch nationalities lacked representation in our study population as well as those who had no experience with the STI clinic’s SHS. This could compromise the external validity of the study results. Due to the hidden and complex nature of the MSW-MSM population, we also have to assume that a part of the population will always remain hidden to care. Given the sensitive nature of the interview topics, the participants may have given socially desirable answers. Due to local differences, our study results might not be generalizable for the entire Western-European region. However, due to cross-border sex work activity the MSW-MSM subculture appears similar in Germany and Belgium and thus the results seem generalizable to those regions and other non-high-urban areas in Western Europe.

## Conclusions

In conclusion, this study has been able to assess characteristics of the MSW-MSM population, identify perceived barriers and facilitators of MSW-MSM to utilise SHS and develop implications to increase accessibility and utilisation of SHS for MSW-MSM. The MSW-MSM population’s diversity and identified barriers, facilitators and implications should be taken into account in sexual healthcare strategies to tailor these to MSW-MSM and optimize accessibility and utilisation of SHS in Western Europe. Additionally, SHS-providers could facilitate sex work disclosure by explicitly and personally asking patients about sex in exchange for money or goods in a non-judgmental manner and explaining the medical relevance of disclosure.

As this is both in Western Europe and globally one of the few studies focusing on SHS utilisation by MSW-MSM, it remains important that future studies further explore the topic of MSW-MSM healthcare accessibility and utilisation and develop and assess STI/HIV prevention programs targeting MSW-MSM.

## Supplementary Information

**Additional file 1.** Study context and study population context. A description of the study context and the research team involved in the study, as well as qualitative results about the context of the sex worker study population.

## Data Availability

The data generated and analysed during the current study are not publicly available due to the possibility of compromising individual privacy but are available from the corresponding author on reasonable request.

## Abbreviations

COREQ: consolidated criteria for reporting qualitative studies
FSW: female sex workers
HBM: Health Belief Model
HBV: hepatitis B virus
HIV: human immunodeficiency virus
IFW: internet fieldwork
MSM: men who have sex with men
MSW-MSM: male sex workers who have sex with men
PrEP: pre-exposure prophylaxis
RAAM: Reasoned Action Approach Model
SHS: sexual healthcare services
STI: sexually transmitted infections
