# Supplementary material for: Barriers and facilitators to utilisation of public sexual healthcare services for male sex workers who have sex with men (MSW-MSM) in The Netherlands: a qualitative study
Source: BMC Public Health. 2022 Jul 21;22:1398. doi: 10.1186/s12889-022-13799-1 (PMC9306090; doi:10.1186/s12889-022-13799-1)
Supplement: Supplementary file 1 — Additional file 1. Study context and study population context. A description of the study context and the research team involved in the study, as well as qualitative results about the context of the sex worker study population. [file 12889_2022_13799_MOESM1_ESM.docx]

**Supplement 1. Study context and study population context**

**Study context**

The research team comprised of both academic scholars and medical professionals with extensive prior experience in the sexual health field working at the Public Health Service South Limburg. One of the SHS-providers in the Netherlands is the Public Health Service’s outpatient STI clinic (hereafter named: STI clinic). The South Limburg STI clinic has three locations in the region. The STI clinic provides free of charge, anonymous and confidential SHS to STI high-risk groups, including sex workers. The SHS include provision of STI/HIV testing, hepatitis B vaccinations, pre-exposure prophylaxis (PrEP) and sexual health counselling. One can visit the STI clinic if one has a Dutch residential address. The province of Limburg is a non-high-urban region with sex work prevalence similar to other non-high-urban regions in The Netherlands and Western Europe.

The interviews were conducted by two female researchers, a researcher with prior qualitative sexual health research experience and a female STI clinic nurse who was also involved in the participant recruitment. Participants were informed of the interviewers’ professions, their experiences with the sex work field and sexual health, the reasons for conducting the interviews and the main study goal.

**Study population context**

*Start sex work*

The majority started spontaneously doing sex work, often by being offered money in exchange for sex at a young age at parties or on online dating platforms Bullchat or Grindr.

*“I was just online and eh, it was one of the first times on Bullchat and then you get a lot of messages right away. And eh, elderly to catch your attention or to persuade you to do that, they usually start offering you money quickly.” – P4, 20y*

*Reason sex work*

Reasons for doing sex work were mainly money-related. Almost all participants did sex work either to earn some extra money or to make a living.

Sexual tension, sexual curiosity and sexual pleasure were important secondary reasons to do sex work. Sex work was thus often seen as a win-win situation. A few participants mainly did sex work in exchange for drugs for chemsex purposes.

*“But if I really think, I can't make it through the month, then it's like, if I get an offer, I can take it if I think, okay, he looks good. Or not too old.” –* P14, 18y

*Social support sex work*

A large part of the study population did not have any form of social support for their sex work. Due to individualization and the discrete nature of the sex work, there was also a lack of peer support, with more than half of the study population not knowing any MSM-MSW colleagues. Some of these participants did not want or need to discuss and disclose their sex work with their social environment, but many would however like to receive social support from the STI clinic and be able to contact them for problems related to sex work. Partly for this reason, participants also expressed a need for a permanent contact person within the STI clinic, most likely a nurse, who is informed about the background of the MSW-MSM. A few sex workers, both with and without social support, needed a sex workers support group, provided it includes sex workers with similar situations.

*“With my tester. I want to talk about uh, when something's wrong, when I don't feel comfort. If I, uh, something happened, I actually want to talk to my tester. To advise me to give me... Because you can't talk to everyone, can you?*“ – P19, 35y
